# Complete genome sequence data of *Pseudomonas nitroreducens* L4, an endophyte isolated from cotton plants

**DOI:** 10.1016/j.dib.2024.110639

**Published:** 2024-06-13

**Authors:** Haiyang Liu, Lubo Zhuang, Qingchao Zeng

**Affiliations:** a Institute of Plant Protection, Xinjiang Academy of Agricultural Sciences, Urumqi, 830091, China; b MOA Key Lab of Pest Monitoring and Green Management, Department of Plant Pathology, China Agricultural University, Beijing 100193, China; c Institute of Plant Protection, Beijing Academy of Agriculture and Forestry Sciences, Beijing 100097, China

**Keywords:** *Pseudomonas nitroreducens*, Biocontrol, Genome sequence, Cotton verticillium wilt, Endophyte

## Abstract

*Pseudomonas nitroreducens* L4 was isolated from the interior of cotton plants, which showed strong biocontrol activity against *Verticillium dahlia* and other fungal pathogens. To elucidate the biocontrol mechanism, the genome sequence of L4 was sequenced using the Illumina and Nanopore sequencing platform. The assembled genome of L4 consisted of a single circular chromosome was 6,229,472 bp, with an average GC content of 64.95 %, 5,629 protein-coding genes, 72 tRNA, 16 rRNA and 1 tm RNA. Six secondary metabolite biosynthetic gene clusters are identified in the genome. The genome sequence provided a theoretical basis for analyzing the biocontrol mechanism of this strain.

Specifications Table

**Table:** 

Subject	Microbiology: Applied Microbiology
Specific subject area	Molecular biology
Data format	Raw and analyzed
Type of data	Figures, Tables
Data collection	The DNA extraction of *P. nitroreducens* L4 using a SteadyPure bacterial genomic DNA extraction kit. The DNA was transferred to Guangdong Magigene Technology Co., Ltd. (Guangzhou, China) to perform the genome sequencing using the Illumina and Oxford Nanopore Technologies.
Data source location	*P. nitroreducens* L4 was isolated from the cotton plants
Data accessibility	The genome sequence of *P. nitroreducens* L4 has been deposited in DDBJ/ENA/GenBank under the accession number CP120376 (https://www.ncbi.nlm.nih.gov/nuccore/CP120376.1)

## 1 Value of the Data

• The genome data of *P. nitroreducens* L4 may be helpful in understanding biological traits related to biocontrol against plant pathogens.
• The genome sequence of *P. nitroreducens* L4 provides fundamental knowledge of this organism and insight for biotechnological application in agriculture.
• The genome data of *P. nitroreducens* L4 will provide valuable information to perform comparative genomics analysis.

## 2 Background

Cotton Verticillium wilt, mainly caused by a soil-inhabiting fungus *Verticillium dahlia*, could seriously damage the yield and quality of cotton [1]. The fungus invades from the roots and systematically infects the whole plants and propagates in xylem vessel. In addition, the microsclerotia, dormant structures formed by *V. dahlia*, play crucial roles in disease spread and its long-term survival in nature [[2], [3]]. Therefore, it is extremely difficult to control cotton Verticillium wilt. It is difficult to obtain Verticillium wilt disease-resistant cotton varieties by traditional breeding methods. Due to a lack of effective sources of resistance, no highly resistant varieties have yet been successfully bred [[1], [2]]. Although chemical fungicides are effective, but they are not environmentally friendly. Meantime, the repeated use of chemicals generates development of resistance in the pathogen and has a negative effect on some beneficial organisms [4]. With the growing public interest in eco-friendly control methods, biological control is currently one of the most promising methods to control cotton Verticillium wilt. The *Bacillus, Streptomyces,* and *Pseudomonas* have documented biocontrol activities against cotton Verticillium wilt [[5], [6], [7]]. Endophytic microorganisms are referred to as the microbes that inhabit the internal parts of plants. The endophyte displayed that it could suppress the pathogen and trigger plant resistance and compete the niche with the pathogen [[8], [9], [10], [11]]. The advantage that endophytes have over other biocontrol agents is the ability to colonize plant's internal tissues which make the endophytes as the potential natural resources for biological control.

## 3 Data Description

**Fig. 1:**
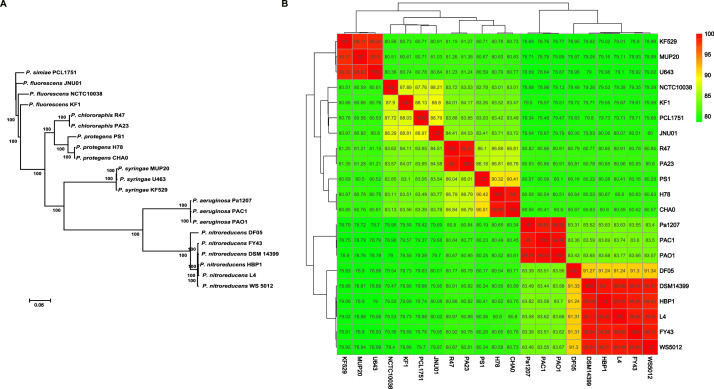
Phylogenetic analysis of *P. nitroreducens* L4. The ML tree of different *Pseudomonas* strains was generated based on 1,800 single-copy core genes using RAxML 8.2.10. Percent bootstrap values (from 100 replicates) are indicated at the nodes (A). Heat-map of ANI values among different *Pseudomonas* strains. The numbers represent the size of ANI values (B).

The complete genome of L4 consists of a single circular chromosome of 6,229,472 bp with a mean G+C content of 64.95 %. In total, 5,718 genes were identified, including 5,629 coding sequences genes (CDSs), 72 tRNA, 16 rRNA and 1 tm RNA genes. The general features were shown in Table 1. Among the predicted CDSs, 3,828 of them could be assigned a putative function, whereas 1,801 were predicted to encode hypothetical proteins. The average length of protein-coding genes is 986 bp. The protein-coding genes account for 89.09 % of the genome sequence (Table 1). Meantime, the phylogenetic tree of the *Pseudomonas* genomes was constructed based on the concentration of the 1,800 core genes that were present in single copy in all genomes with maximum likelihood (ML) methods and rooted by *P. simiae* PCL1751. As shown in Fig. 1, the strain L4 was found in the same clade with other *P. nitroreducens* strains and a sister group of *P. nitroreducens* HBP1 (Fig. 1). Based on Average Nucleotide Identity (ANI) values, the genome sequence of L4 displayed highest similarity with the *P. nitroreducens* strains with the ANI values over 97 %, whereas the ANI values between LQ-3 and other *Pseudomonas* strains were lower, and ranged between 78.98 % and 91.31 % (Fig. 1). Meanwhile, the clustering analysis based on ANI values among each strain showed that *P. nitroreducens* strains clustered together. The heatmap analysis based on ANI values of different *Pseudomonas* strains confirmed the findings of the phylogenetic analysis. But the phylogenetic analysis of *P. nitroreducens* need to further confirm. The genome of L4 was subjected to an automated search using antiSMASH (version 7.0), six secondary metabolism gene clusters were identified (Table 2). Based on the results of the genome analysis, we need to perform the functional analysis of the gene clusters using the molecular biology to find out whether one or more of these clusters contribute to the antimicrobial activity of strain L4 to the cotton Verticillium wilt and other plant pathogens. Then, we can better promote the filed application. The complete genome sequence of L4 will help understand the genetic and genomic diversities of *P. nitroreducens*. It could also represent a valuable resource for the study of biological control functions and plant-microbe interactions.

**Table 1:** General genome features of strain *P. nitroreducens* L4.

Attribute	Values
Genome size (bp)	6,229,472
GC content (%)	64.95
Protein coding genes	5629
RNA genes	89
rRNA	16
tRNA	72
ncRNA	1
Gene total length (bp)	5,578,673
Average length of protein-coding genes (bp)	986

**Table 2:** Secondary metabolite gene clusters in *P. nitroreducens* L4 predicted by antiSMASH 7.0.

Cluster	Type	Most similar known cluster	MIBiG accession
Cluster 1	betalactone	corynecin III/corynecin I/corynecin II	BGC0002284
Cluster 2	ranthipeptide	Pf-5 pyoverdine	BGC0000413
Cluster 3	RiPP-like	-	-
Cluster 4	redox-cofactor	lankacidin C	BGC0001100
Cluster 5	NRP-metallophore	crochelin A	BGC0002001
Cluster 6	NAGGN	-	-

## 4 Experimental Design, Materials, and Methods

In our previous study, strain L4 was obtained from the interior of cotton plants. It has been identified as *P. nitroreducens* by 16S ribosome RNA (rRNA) gene phylogenetic analysis [12]. Our results also demonstrated that strain L4 showed significantly inhibition to cotton Verticillium wilt on plate and pot experiments [12]. Strain L4 was cultured in Luria Bertani (LB) liquid medium at 37°C, with 200 rpm shaking, for 48 h. Then, bacterial culture was harvested for DNA extraction using a *SteadyPure* bacterial genomic DNA extraction kit (Accurate Biotech, Hunan) following the manufacturer's protocols. The quality and quantity of the total DNA was evaluated by Nanodrop One, 0.38 % agarose gel electrophoresis and Qubit 3.0 Fluorimeter (Thermo Fisher Scientific, USA), respectively. Then, the DNA was transferred to Guangdong Magigene Technology Co., Ltd. (Guangzhou, China) to perform the genome sequencing. Whole genome sequencing was performed using the Illumina and Oxford Nanopore Technologies (ONT). For Illumina sequencing, 1 µg of genomic DNA was sheared using the Covaris instrument. The DNA fragments of 500 bp in length was selected using 1.8 % agarose gel and sequencing libraries were produced using ALFA-SEQ DNA Library Prep Kit (Illumina). Sequencing was performed on Illumina NovaSeq 6000 and 150 bp paired-end reads were generated. Reads quality were checked with Sickle v1.33 (https://github.com/najoshi/sickle) with default settings and low quality reads were removed. For long-read Nanopore sequencing, a genomic library was prepared using the Nanopore ligation sequencing kit (SQK-LSK109; Oxford Nanopore, Oxford, UK). Library quality was detected by Qubit 4.0 (Life Technology, USA) and average fragment size was estimated on an Agilent 4200 (Agilent, USA). Finally, the library was sequenced on an Nanopore MinION. Barcode and adapter sequences from Nanopore long reads were trimmed using Porechop v0.2. (https://github.com/rrwick/Porechop) for downstream analysis. After quality control, ∼2.12 G and ∼1.48 G of clean data were collected, respectively. The high-quality short-read and long-read sequences were assembled into a complete sequence using Unicycler v.0.4.9 with default setting [13]. The highly accurate Illumina short reads were aligned against the long Nanopore reads to sort out random sequencing errors [13]. Genomic G+C content and assembly statistics were determined using own Perl script [5]. Gene predictions were performed with Prokka version 1.11 which predicts coding DNA sequence (CDS) using Prodigal [14]. Annotation of the protein-coding sequence was conducted using the Basic Local Alignment Search Tool (BLAST) against the COG, Kyoto Encyclopedia of Genes and Genomes, and Interpro databases [10]. Analysis of secondary metabolite biosynthetic gene clusters was done with antiSMASH [15]. Additionally, the average nucleotide identity was calculated using online JSpeciesWS software (https://jspecies.ribohost.com/jspeciesws/).

## Limitations

Not applicable.

## Ethics Statement

The current work does not involve human subjects, animal experiments, or any data collected from social media platforms.

## CRediT authorship contribution statement

**Haiyang Liu:** Conceptualization, Data curation, Formal analysis, Funding acquisition, Investigation, Methodology, Project administration, Resources, Software, Supervision, Validation, Visualization, Writing – original draft, Writing – review & editing. **Lubo Zhuang:** Conceptualization, Formal analysis, Investigation, Methodology, Project administration, Resources, Software, Supervision, Validation, Visualization, Writing – review & editing. **Qingchao Zeng:** Conceptualization, Data curation, Investigation, Methodology, Project administration, Resources, Software, Supervision, Validation, Visualization, Writing – original draft, Writing – review & editing.

## Data Availability

NCBI (Original data) (Mendeley Data).
